# The evolution of pyrimethamine resistant *dhfr *in *Plasmodium falciparum *of south-eastern Tanzania: comparing selection under SP alone *vs *SP+artesunate combination

**DOI:** 10.1186/1475-2875-10-317

**Published:** 2011-10-26

**Authors:** Allen L Malisa, Richard J Pearce, Ben M Mutayoba, Salim Abdullah, Hassan Mshinda, Patrick S Kachur, Peter Bloland, Cally Roper

**Affiliations:** 1 Sokoine University of Agriculture (SUA), Department of Biological Sciences, Faculty of Science, Box 3038, Morogoro, Tanzania; 2 Ifakara Health Institute (IHI), Ifakara, Kilombero District, Morogoro, Tanzania; 3 London School of Hygiene and Tropical Medicine, Pathogen Molecular Biology Unit, Department of Infectious Tropical Diseases, Keppel Street, London, WC1E 7HT, UK; 4 Sokoine University of Agriculture (SUA), Department of Veterinary Physiology, Biochemistry, Pharmacology and Toxicology, Faculty of Veterinary Medicine, PO Box 3017, Morogoro, Tanzania; 5 Malaria Epidemiology Branch, Division of Parasitic Diseases, National Center for Infectious Diseases, Centers for Disease Control and Prevention (CDC), Atlanta, GA, USA

**Keywords:** Evolution, pyrimethamine resistance, microsatellites, combination therapy

## Abstract

**Background:**

Sulphadoxine-pyrimethamine (SP) resistance is now widespread throughout east and southern Africa and artemisinin compounds in combination with synthetic drugs (ACT) are recommended as replacement treatments by the World Health Organization (WHO). As well as high cure rates, ACT has been shown to slow the development of resistance to the partner drug in areas of low to moderate transmission. This study looked for evidence of protection of the partner drug in a high transmission African context. The evaluation was part of large combination therapy pilot implementation programme in Tanzania, the Interdisciplinary Monitoring Programme for Antimalarial Combination Therapy (IMPACT-TZ)

**Methods:**

The growth of resistant *dhfr *in a parasite population where SP Monotherapy was the first-line treatment was measured for four years (2002-2006), and compared with the development of resistant *dhfr *in a neighbouring population where SP + artesunate (SP+AS) was used as the first-line treatment during the same interval. The effect of the differing treatment regimes on the emergence of resistance was addressed in three ways. First, by looking at the rate of increase in frequency of pre-existing mutant *dhfr *alleles under monotherapy and combination therapy. Second, by examining whether *de-novo *mutant alleles emerged under either treatment. Finally, by measuring diversity at three *dhfr *flanking microsatellite loci upstream of the *dhfr *gene.

**Results:**

The reduction in SP selection pressure resulting from the adoption of ACT slowed the rate of increase in the frequency of the triple mutant resistant *dhfr *allele. Comparing between the two populations, the higher levels of genetic diversity in sequence flanking the *dhfr *triple mutant allele in the population where the ACT regimen had been used indicates the reduction in SP selection pressure arising from combination therapy.

**Conclusion:**

The study demonstrated that, alleles containing two mutations at the *dhfr *have arisen at least four times independently while those containing triple mutant *dhfr *arose only once, and were found carrying a single unique Asian-type flanking sequence, which apparently drives the spread of pyrimethamine resistance associated *dhfr *alleles in east Africa. SP+AS is not recommended for use in areas where SP cure rates are less than 80% but this study reports an observed principle of combination protection from an area where pyrimethamine resistance was already high.

## Background

Artemisinin-based combination therapy (ACT) is the World Health Organization (WHO) recommended anti-malarial in areas where SP failure prompts replacement. Already, Tanzania has replaced SP with a combination of artemether and lumefantrine (Coartem^®^). However, SP remains the only option for intermittent treatment of malaria during pregnancy and an important drug in the intermittent treatment of infant. Therefore, priority remains for continuing surveillance and monitoring of genetic changes of SP resistance in the population.

SP kills parasites by inhibiting folate biosynthetic pathway [1-3]. Pyrimethamine competitively inhibits the enzyme dihydrofolate reductase, DHFR [1] while sulphadoxine does the same to inhibit the enzyme dihydropteorate synthase, DHPS [2,3]. Resistance to SP is associated with point mutations in the genes coding for the two enzymes, *dhfr *and *dhps *[4-8]. Single mutation at codon 108 (S108T) confer low level of insensitivity to the drug [5]. Additional mutations increases the insensitivity of the parasite to the drug; the double mutation (N51I+S108N) or (C59R+S108N) confers intermediate resistance [5,9] increasing to triple mutations (N51I+C59R+S108N). An additional mutation at codon 164 (I164L) leading to quadruple mutant, cause highest level of resistance to SP. It is now known that, quadruple mutation exist in Africa in a very low frequency [10-13, this study], although recent study in Uganda [14] indicates its frequency is increasing.

There is currently conflicting information about how SP resistant alleles originate and spread across populations [13-20]. Early studies suggest that, the *dhfr *triple mutant allele is monophyletic in origin indicating it has arisen once [15] and subsequent studies reported the lineage had arisen in south-east Asia [16]. Although many studies that followed confirmed this observation [14,17-20], a study in an area of high malaria transmission in western Kenya identified presence of multiple lineages of the *dhfr *triple mutant allele [13], and concluded that, local evolutionary history as well as dispersal by gene flow are equally important in the establishment of resistance in populations.

This study has investigated the rate of selection and evolution of *dhfr *resistant alleles in two populations with contrasting anti-malarial therapies, Kilombero-Ulanga under SP monotherapy and Rufiji under SP+AS. By using three sets of microsatellite loci flanking the *dhfr *gene, the study investigated the evolutionary origin of resistant *dhfr *alleles and compares the rate of underlying genetic exchange between the two populations.

## Methods

### Study area, subjects and samples

Samples were collected through three community cross sectional surveys conducted during July-September in 2001, 2002 and 2006 in three rural districts of south-eastern Tanzania; Rufiji (Population = 203,000), Kilombero (Popupulation. = 322,000) and Ulanga (Population = 194,000). The surveys were part of large combination therapy pilot implementation programme in Tanzania, the Interdisciplinary Monitoring Programme for Antimalarial Combination Therapy (IMPACT-TZ). IMPACT-Tanzania is a multiyear implementation research evaluation that rests on a collaborative platform incorporating the US Center for Disease Control and Prevention (CDC), the Ifakara Health Institute (IHI), London School of Hygiene and Tropical Medicine (LSHTM), and the Ministry of Health and Social Welfare including its National Malaria Control Programme, the Tanzania Essential Health Interventions Project and the Council Health Management Teams of Rufiji, Kilombero, Ulanga, Morogoro and Mvomero Districts. For the purpose of the study, Kilombero and Ulanga Districts were treated as a single district because population movement between these two districts is high and the study population spans the border region. *Plasmodium falciparum *malaria transmission in the study area was intense (with an estimated entomological inoculation rate of 367 infectious bites per person per year [21] and perennial with some seasonal fluctuation (however, recent studies indicate decrease in malaria transmission in all endemic areas but new entomological inoculation rate for the studied area has not yet been re-determined). The survey conducted in Jul-Sept 2001 coincided with the implementation of new national policy replacing CQ with SP as the recommended first-line treatment of uncomplicated malaria. In 2002 the timing of the survey coincided with the implementation of SP+AS combination in Rufiji. While the national policy change to SP in 2001 was implemented nationwide, the change from SP to SP+AS combination in 2002 was applied in Rufiji district only.

A total of 22,696 adults and children belonging to randomly selected households participated in the study (6,482, 7,447 and 8,767 in 2001, 2002 and 2006 survey, respectively), and a finger-prick blood sample for blood slide and filter paper bloodspot were collected from each individual. The filter paper bloodspots were air-dried and stored at room temperature in self-sealing plastic bags with desiccant and stored at room temperature for future use in genetic testing. All blood slide samples were screened by microscopy for *P. falciparum *positivity. Bloodspots from microscopically positive subjects were selected for molecular genotyping.

### Ethics

Scientific and ethical clearance was granted from the Medical Research Council of the National Institute for Medical Research in Tanzania, the Centre for Disease Control and Prevention, USA, and the London School of Hygiene and Tropical Medicine, UK. Consent was obtained from all individuals or their guardians before collection of samples.

### DNA extraction

The DNA was extracted from bloodspots dried on filter papers. A sector of the dried blood spot filter paper was excised using a sterile blade or scissors, and soaked in a 1 ml, 0.5% saponin-1x phosphate buffered saline (PBS) overnight in a 96-deepwell plate. The segment was then washed twice in 1 ml of 1x PBS and finally, was boiled for 8 min in 100 ul PCR quality water with 50 ul 20% chelex suspension (pH 9.5).

### PCR amplification

Nested PCR was used to amplify a 594 bp fragment of *dhfr *containing the sequence where mutations are found. Primer sequences and PCR reaction conditions were previously described in [22] PCR was performed in 96 well plates with 25-μl PCR reaction volumes containing final concentrations of 0.25 μM oligonucleotide primers, 2 mM MgCl_2_, 250 μM each deoxyribonucleotide triphosphate (dNTPs), and 1x Taq polymerase. One microlitre (1 μl) of DNA template was used in the outer (primary) PCR reaction mixture. The outer *dhfr *PCR products were diluted three fold before a 1 ul was introduced into the inner PCR reaction mixtures.

### Molecular genotyping of point mutations by Sequence Specific Oligonucleotide Probing (SSOP)

The amplified PCR products were screened for *dhfr *sequence variants at 5 loci where single nucleotide polymorphisms (SNPs) are known. The sequence changes (and the amino acid substitutions they code for) are summarized in Table 1.

**Table 1:** The nucleotide and amino acid substitutions at *dhfr *gene screened for by PCR-SSOP

Codon	50	51	59	108	164
Wild type	Cys (C) TGT	Asn (N) AAT AAC	Cys (C) TGT	Ser (S) AGC	Ile (I) ATA

Mutant	**Arg (R)** **CGT**	**Ile (I)** **ATT**	**Arg (R)** **CGT**	**Asn (N) AAC** **Thr (T)** **ACC**	**Leu (L)** **TTA**

PCR products were spotted in a 12 by 8-grid and cross linked onto nylon membranes and probed for sequence polymorphisms by hybridisation to specific oligonucleotide probes described previously [21]. The probed blots were visualized using ECF substrate and detection using a phosphorimager (STORM). Inspection of the phophorimager output was recorded through viewing of digitally captured images of chemi-fluorescent signal.

The stringency and specificity of the hybridisation process was confirmed by inspection of a series of four controls of known single genotype variant sequence. All blots with non-specifically bound probes were stripped and re-probed. A SNP was considered to be present in the PCR product when the intensity of signal was higher than that of the background. The blots were scored independently by two people and the results reconciled.

The study aimed to establish the relative abundance of different point mutation haplotypes at *dhfr*. Since bloodstage *P. falciparum *is haploid this is very straightforward when an infection consists of a single genotype because only one form of sequence is seen at every SNP locus. When infections are composed of multiple genotypes a mixture of different sequence variants occur making the inference of point mutation haplotypes within that infection impossible.

The presence, absence, and relative abundance of hybridisation signal were recorded for every probe at each locus. A sample was considered to have a single haplotype when only one sequence variant was found at each locus. Blood samples were categorized as having a single, a majority or mixed form of sequence at every SNP locus. Majority and mixed genotype infections were differentiated according to the relative intensity of signal. To determine the relative abundance of different point mutation haplotypes in the parasite population one haplotype only was counted from each infection and those mixed infections where haplotypes could not be resolved were omitted from the calculation of haplotype frequencies. Hence frequency data is based upon a subset of isolates, which were unmixed or had a predominating majority haplotype. A breakdown of the proportions of isolates which PCR amplified and which were genotyped as single, majority or mixed haplotype infections obtained for the 2006 annual survey is given in Table 2.

**Table 2:** Proportion of samples that were *P.falciparum *positive, PCR amplified *dhfr*, and single/majority *dhfr*

Year	Rufiji			Kilombero Ulanga		
	
	2001	2002	2006	2001	2002	2006
Survey population	3285	3349	4267	3197	4098	4500

*P. falciparum *positive	908 (27.6%)	854 (25.5%)	916 (21.5%)	580 (18.1%)	875 (21.3%)	645 (14.3%)

PCR amplified *dhfr*	683	687	683	488	686	294

Single or majority *dhfr*	420	527	616	238	489	275

### Microsatellite analysis

In order to analyse microsatellite immediately flanking sequence polymorphism on chromosomes carrying specific allelic forms of *dhfr*, the study used the genotype data obtained by PCR-SSOP method described above to identify and locate DNA sample bearing that particular allelic form. For the purpose of looking on these flanking sequence polymorphisms, the study deliberately selected a subset of samples that were unmixed at any polymorphic loci at *dhfr *(i. e. samples in which only single haplotype were obtained). Nonetheless, two or more alleles were still detected in some of the samples and such samples were classified as polyclonal and hence were excluded from haplotype construction and any further analysis. Microsatellite sequence located 0.3 kb, 4.4 kb, and 5.3 kb upstream of codon 108 of the *dhfr*-ts gene, which is on chromosome 4, were analysed. A semi nested PCR system employing the primer sequences and PCR cycling parameters described in [15] was used. A total of 728 samples comprising of 126 *dhfr *wild type (N51+C59+S108), 346 double mutant *dhfr *(N51I+S108N or C59R+ S108N) and 256 triple mutant *dhfr *(N51I+ C59R+ S108N) were selected from 2001, 2002 and 2006 genotypic data. Microsatellite PCR samples were diluted 1:100 and run with LIZ-500 size standard on an ABi 3730 genetic analyser (Applied Biosystems, Warrington, Cheshire, UK). Allelic sizes were separated on an Applied Biosystems ABi 3730 capillary sequencer and were scored using Gene Mapper software version 3.7 (Applied Biosystems, Warrington, Cheshire, UK).

### Statistical analysis

Statistical comparison of allele frequencies at *dhfr *was carried out using Fishers exact test in STATA version 9.2. The calculation of binomial exact 95% confidence intervals was carried out using STATA version 9.2 [23]. Gene diversity values was calculated as He = [n/(n-1)][1- ΣPi^2^] where n is the number of samples and Pi is the frequency of the ith allele.

## Results

Of 22,696 people sampled, 4,778 were found to be infected with *P. falciparum*. DNA was extracted from the 4,778 *P. falciparum *positive bloodspots and PCR amplification of *dhfr *performed once, giving a combined rate of PCR amplification success of 74% (Table 2). The amplified products were screened for all the variant sequences described in Table 1. Out of the 3,521 isolates which amplified successfully for *dhfr*, 2,565 (80%) were single or majority genotype infections and the point mutation haplotypes could easily be resolved.

**Figure 1:**
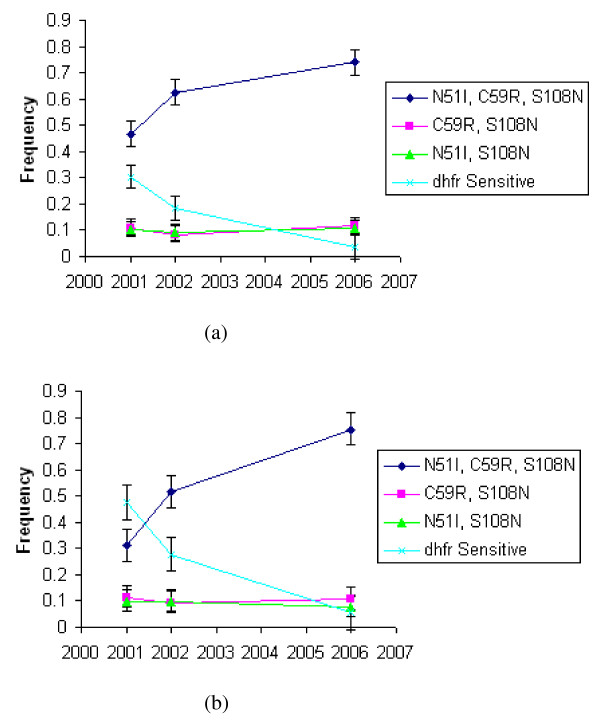
**Allelic haplotype frequency change at *dhfr *gene over time: (a) Rufiji (b) Kilombero - Ulanga**. Frequency values are shown with 95% CI bars calculated by binomial exact method.

Point mutations were detected at codons 51, 59, 108 and 164 of the *dhfr *gene. The same mutations with the exception of codon 164 was reported in previous studies in Tanzania [15,22,24,25] and are common throughout east Africa, Malawi [26], Kenya [27,28] and Uganda [29,30]. These point mutation occurred in four haplotypic configurations at *dhfr *(sensitive, N51+C59+S108, double mutant, N51I+S108N or C59R+S108N, and triple mutant, N51I+C59R+S108N). Rare haplotypes were also detected at *dhfr *gene (single mutants, S108N and C59R) in each case the frequency of the haplotype was < 4%. Mutation at codon 164 (I164L) was detected in only one of the 3,521 isolates that amplified at *dhfr *gene, and it occurred as a mix of minority mutant in a sensitive majority. Figure 1 compares change in frequency of allelic haplotypes at *dhfr *over time, in Rufiji (implementing SP + AS combination, Figure 1a) and Kilombero-Ulanga (implementing SP monotherapy, Figure 1b).

**Figure 2:**
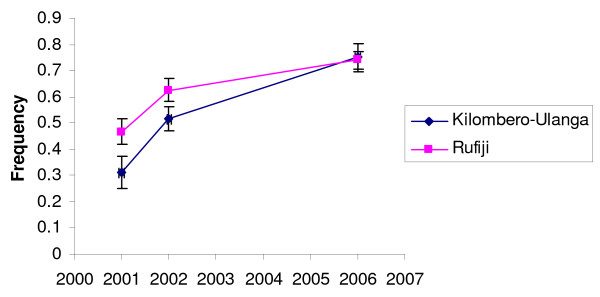
**Comparison of *dhfr *triple mutant allelic haplotype between Kilombero-Ulanga and Rufiji populations in 2001, 2002 and 2006**. Frequency values are shown with 95% CI bars calculated by binomial exact method.

A similar trend of the *dhfr *allelic frequency change over time was observed both in Kilombero-Ulanga and Rufiji populations, indicating that ACT in Rufiji had little and non significant effect on the increase of resistant *dhfr *alleles. Most striking is the effect of national policy change from CQ to SP first-line treatment in late 2001, leading to significant rapid increase of triple mutant *dhfr *allele in both districts (P ≤ 0.0001), displacing the sensitive *dhfr *allele as described in detail elsewhere [25]. Changes in the frequency of the *dhfr *triple mutant allele (N51I+C59R+S108N) are shown in Figure 2. Its frequency was significantly higher in Rufiji than in Kilombero-Ulanga between 2001 and 2002, yet the increase of this allele during 2001- 2002 period was highly significant in both Kilombero-Ulanga (p ≤ 0.0001) and Rufiji (p ≤ 0.0001). Although at the start of SP+AS combination in Rufiji in late 2002 the frequency of triple mutant *dhfr *allele was higher in Rufiji (63%) than Kilombero-Ulanga (52%), the ACT slowed its rate of increase in Rufiji where by 2006 the frequency was higher in Kilombero-Ulanga (75%) than Rufiji (74%).

In 2001, the frequency of the sensitive *dhfr *haplotype (N51+C59+S108) was significantly higher in Kilombero-Ulanga (48%) than Rufiji (30%) (P ≤ 0.0001). As the triple mutant *dhfr *allele increased in both districts, the sensitive *dhfr *allele was displaced proportionately reducing its frequency to (6%) in Kilombero-Uanga and (4%) in Rufiji (P ≥ 0.05) by 2006. The N51I+S108N and C59R+S108N double mutant *dhfr *alleles persisted throughout unchanged with an average frequency of roughly 10% at both populations, indicating they are independent of drug pressure (Figure 1).

The evolutionary origins of *dhfr *haplotypes was assessed by genotyping 728 isolates (CNCS = 126, CNRN + CICN = 346, and CIRN = 256) of *P. falciparum *carrying resistant or the sensitive *dhfr *alleles. Although all these samples were unmixed based on the PCR-SSOP point mutation genotype data, 99 (14%) isolates contained > 1 allele at one or more microsatellite loci, indicating existence of multiple *P. falciparum *clones while also highlighting higher sensitivity of microsatellite in detecting polyclonality compared to PCR-SSOP method. A further 183 (25%) isolates did not amplify at one or more microsatellite loci making construction of haplotype incomplete. Both the polyclonal, and isolates with incomplete microsatellite data were excluded from haplotype construction and any further analysis. Overall, 446 (61%) isolates, were included in haplotype construction and analysis of diversity values; 78 were the *dhfr *sensitive, 184 double mutant *dhfr *(C59R + S108N or N51I + S108N) and 184 the triple mutant *dhfr *(N51I + C59R + S108N).

Polymorphisms at microsatellite loci were recorded (Table 3). Numerous alleles at the 3 *dhfr *linked microsatellite loci were identified. The -0.3 kb locus had 11 alleles of 85-115 bp, the -4.4 kb locus had 19 alleles of 156-199 bp and the -5.3 kb locus had 18 alleles of 190-221 bp, and these were evenly distributed among the two named districts.

**Table 3:** Number of alleles (A) and allele length range at the three microsatellite loci in Kilombero-Ulanga and Rufiji populations

Locus	Kilombero-Ulanga		Rufiji		Overall	
	
	A	Size range (bp)	A	Size range (bp)	A	Size range (bp)
-0.3	10	85-115	9	87-115	11	85-115

-4.4	17	158-193	17	156-199	19	156-199

-5.3	15	190-219	13	190-221	18	190-221

**Figure 3:**
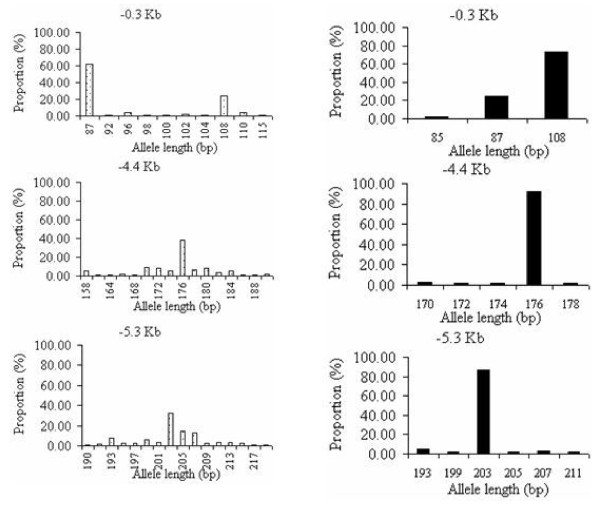
**Frequency and distribution of microsatellite allele at the 3 loci upstream of *dhfr *gene**. Sensitive (white) on the left and Triple mutant *dhfr *(black) on the right.

Frequency of microsatellite alleles flanking the sensitive and triple-mutant *dhfr *allele groups are presented in Figure 3. In the sensitive type *dhfr*, many alleles were detected at each microsatellite locus revealing high diversity in the DNA flanking the sensitive *dhfr *alleles. In the *dhfr *triple-mutant group, at each locus, few low-frequency alleles and a single predominant allele were found in most isolates. These alleles were the 108 bp at -0.3 Kb, 176 bp at -4.4 Kb and the 203 bp at the -5.3 Kb highlighting the extent of lost diversity. The high frequency of allele 108, 176 and 203 reflect the shared ancestry of the triple mutant in these populations while the observed rare alleles are reflection of the recent evolution by mutation or recombination [31,32].

Three-locus microsatellite haplotypes were constructed from the 446 unmixed isolates, in the sensitive, double mutant (C59R+S108N), double mutant (N51I+S108N) and triple mutant *dhfr *groups (see Additional file 1). A total of 104 different haplotypes were identified, ranked and numbered according to size of allele at -0.3 Kb then at -4.4 Kb and finally at -5.3 Kb locus and numbered from H1-H104. Of the 104 different microsatellite haplotypes, 56 were identified among the 78 sensitive isolates, 19 among the 87 C59R+S108N double mutant isolates, 26 among the 97 N51I+S108N double mutant isolates and 21 were identified among the 184 triple mutant *dhfr *isolates. Nine haplotypes (H16, H22, H32, H35, H36, H39, H47, H51 and H53) were shared differently between sensitive and double mutant and triple mutant *dhfr *allele (Additional file 1). Two others (H45 and H52) found among 19 C59R+S108N double mutant isolates were shared with the N51I+S108N double mutant *dhfr*. H90 was in the majority of the triple mutant *dhfr *isolates (135 of 184) but was also found in 2 of 106 among N51I+S108N double mutant *dhfr *isolates. H89 was shared (2 of 106 against 2 of 184) among the N51I+S108N double mutant and the triple mutant *dhfr *isolates, respectively. This large extent of haplotype sharing is probably a reflection of high degree of recombination in this area of stable high malaria transmission and is consistent with a similar finding in Kenya [20]. The findings reported here confirms recent findings from Kilifi Kenya [20], reporting high levels of microsatelite haplotype recombination.

**Figure 4:**
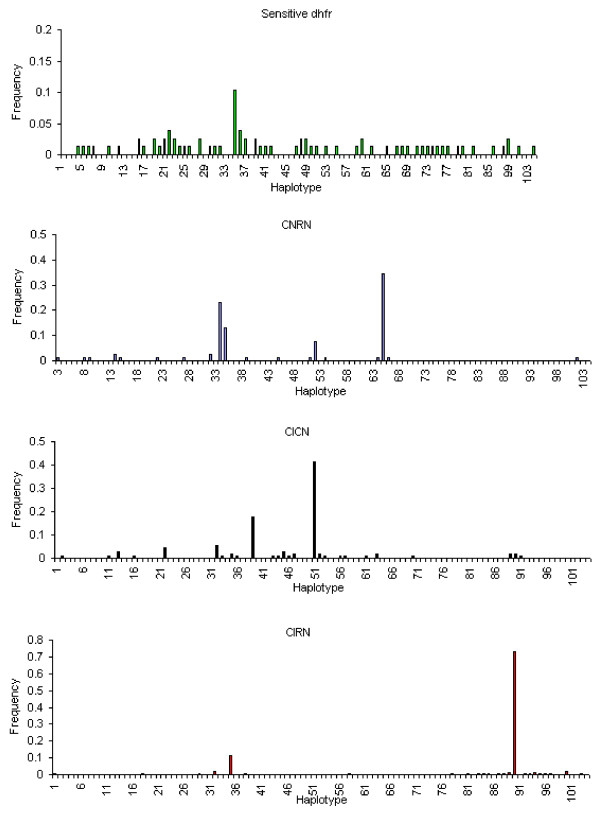
**Microsatellite allele haplotypes in the flanking region upstream of *dhfr***.

There was a clear difference in the haplotypic diversity between the sensitive and all resistant forms of the *dhfr *chromosomes. While the sensitive forms revealed high diversity with all the 56 haplotypes among 78 isolates contributing on average equally to the gene pool, the resistant forms had just a few predominant haplotypes, with the number of dominant haplotypes decreasing with increasing level of resistance (Figure 4). The triple mutant *dhfr *chromosome had a single dominant haplotype (H90) which was identical to the Asian origin type described in [16], and later reported to be wide-spread in Africa; Tanzania, Mozambique and South Africa [17], Senegal [18], Kenya [13,20], Benin, Cameroon, Comoros, Congo-Brazzaville, Ivory coast, Gabon, Ghana, Guinea, Mali, Senegal and Uganda [14,19]. Seventy three percent (135) of the triple mutant *dhfr *isolates analysed here were found to carry this haplotype.

Beside the predominant haplotype H90, the triple mutant *dhfr *had 21 other microsatellite haplotypes, and these differed at one or two of the three flanking microsatellite loci. These alternative haplotypes were generally of low frequency of < 2% (except haplotype 21 which occurred at 11% frequency) and most likely to be relatives of the dominant haplotype with the different microsatellite allele(s) possibly introduced by recombination with the double *dhfr *mutant types. An exception however, is the existence of four haplotypes (H32, H35, H18 and H38), which had the 87 bp allele at the closest *dhfr *microsatellite loci (-0.3 Kb). The 87 bp allele at -0.3 Kb loci is a common allele for the sensitives and double *dhfr *mutants, while 108 bp is for the triple *dhfr *mutants, thus there is a possibility that these haplotypes represent cases where double mutants have acquired an extra mutation transforming to triple *dhfr *mutant types.

Two novel haplotypes (H29 and H58) associated with the triple mutant type were identified and differed by allele length with the dominant (Asian type) haplotype at all three microsatellite loci. They have not been described before and in this study each was present in one isolate only. The haplotypic diversity displayed by the double mutant *dhfr *isolates was intermediate between that found in the sensitive and triple mutant *dhfr *types. Both double mutant *dhfr *alleles were associated with four common haplotypes, the C59R+S108N with H35, H51, H34 and H64 and N51I+S108N with H22, H32, H39 and H51. H64 was the most common for the C59R+S108N double mutant, while H51 was the most common for the N51I+S108N double mutant *dhfr*. Both haplotypes were reported in Tanzania and South Africa [15] and later found in other African *P. falciparum *populations [13,14,17-19].

**Figure 5:**
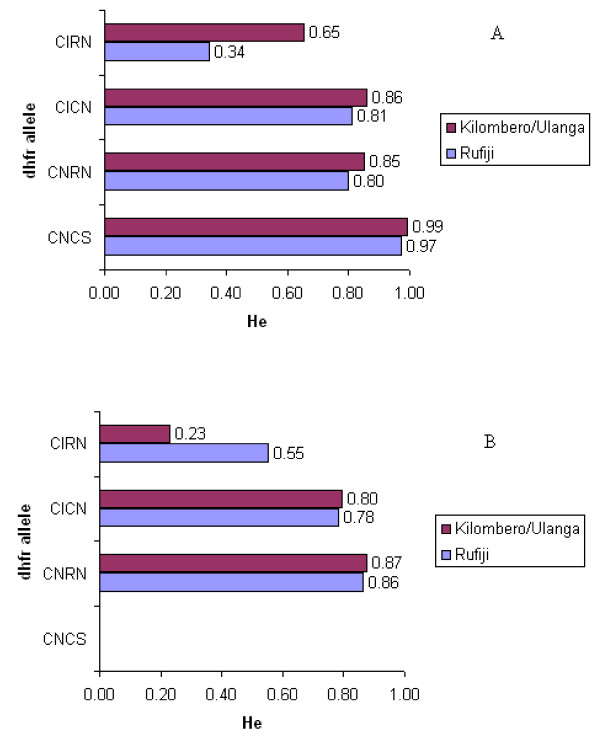
***Dhfr *flanking microsatellite heterozygosity**. A) 2001/02 and B) 2006. Note: in B the CNCS was already too rare to get sufficient samples.

Both *dhfr *double mutant alleles heterozygosity values were almost similar (C59R+S108N; He = 0.800 in 2001/02 and 0.86 in 2006 in Rufiji and 0.85 in 2001/02 and 0.87 in 2006 in Kilombero-Ulanga, and N51I+S108N; He = 0.81 in 2001/02 and 0.78 in 2006 in Rufiji and 0.85 in 2001/02 and 0.79 in 2006 in Kilombero-Ulanga) in both populations indicating little change in diversity over time (Figure 5). To examine if there were any temporal or geographical trends in the extent of diversity flanking the major resistance alleles, isolates from two populations of Kilombero-Ulanga and Rufiji at two time points (2001/02 and 2006) were compared. Generally, it was observed that the common microsatellite haplotypes associated with resistant *dhfr *chromosomes were broadly consistent at both surveys (2001/02 and 2006). Distribution of both common haplotypes and heterozygosity values at two survey points (2001/02 versus 2006) and between the two populations (Kilombero-Ulanga versus Rufiji) indicated that the two populations were broadly similar, and this is most likely attributed to gene flow.

**Figure 6:**
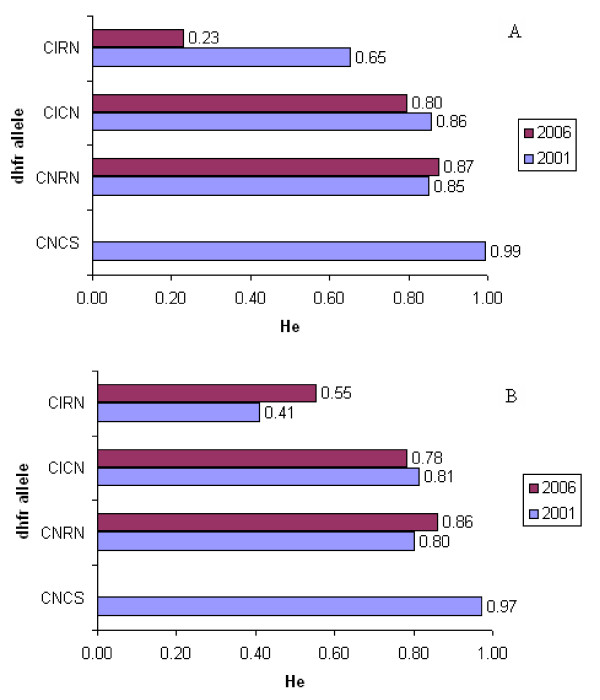
***Dhfr *flanking microsatellite heterozygosity**. A) Kilombero-Ulanga B) Rufiji. Note: in 2006 the CNCS was already too rare to get sufficient samples.

The study also examined the levels of gene diversity, by computing haplotype-based heterozygosity values for each *dhfr *allele (Figure 5). The sensitive *dhfr *allele had highest diversity, He of 0.99 and 0.97 in Kilombero-Ulanga and Rufiji, respectively, reflecting its ancestral state [15]. By contrast, the resistant *dhfr *alleles showed a progressive decrease of diversity with increasing number of mutation; the *dhfr *double mutant C59R+S108N, He = 0.863, 0.8309 and N51I+S108N, He = 0.8262, 0.7978 in Kilombero-Ulanga and Rufiji, respectively. Most striking was the amount of reduced diversity detected in the triple mutant *dhfr *indicating more than a half of heterozygosity had disappeared, He = 0.3419 and 0.4828, in Kilombero-Ulanga and Rufiji, respectively, when compared with sensitive *dhfr *alleles. The heterozygosity values for the resistant *dhfr *alleles between the 2001/02 and 2006 at the two populations of Rufiji and Kilombero-Ulanga were also compared (Figure 6).

The temporal relationship comparing triple mutant *dhfr *allele heterozygosity values between Kilombero-Ulanga and Rufiji populations generated interesting findings. In Rufiji, He was 0.34 in 2001/02, but increased slightly to 0.55 in the 2006 while in Kilombero-Ulanga, He was 0.65 in 2001/02 but decreased dramatically to 0.23 in 2006. These findings indicate that while escalation of SP drug pressure in Kilombero-Ulanga led to dramatic loss of gene diversity, SP+AS in Rufiji was associated with halt of the loss and stabilization of gene diversity of the triple mutant *dhfr *allele. Both the sensitive and double mutant *dhfr *alleles did not seem to respond to either SP or SP+AS drug pressure.

## Discussion

The study indicate that the escalation of *dhfr *triple mutant resistance allele between 2001/02 - 2006 in Kilombero-Ulanga and Rufiji populations coincided with the change of Nationla anti-malarial policy from CQ first line to SP in late 2001/02. In fact, this observation is concordant to the results of SP treatment efficacy monitoring in southeast Tanzania during 2003, which found that 49% of SP treatments failed by day 28 [33]. It is worth noting that, the observed escalation of *dhfr *triple mutant resistance allele in the study districts, was the continuation of already established pyrimethamine resistance, following 18 years of SP use as a second-line treatment since 1983. By the time SP was officially replacing CQ as national first line treatment for uncomplicated malaria, the frequencies of the *dhfr *triple mutant resistance allele were 31% and 47% in Kilombero-Ulanga and Rufiji districts, respectively. As the SP became widely used between late 2001/02-2006, drug pressure intensified and the triple mutant *dhfr *(N51I+C59R+S108N) allele escalated by a > 2-fold increase in Kilombero-Ulanga population. In Rufiji population the SP drug pressure that was initiated by the change of National anti-malarial guideline in late 2001/02 was interrupted by introduction of SP+AS combination in late 2002. It is widely believed that ACT acts so rapidly killing parasite biomass irrespective of their resistant properties, potentially reducing the likelihood that resistant parasites spread within the community as well as potentially reducing overall malaria transmission rates [34,35]. While the combination of SP+AS is not recommended for use in areas where SP cure rates are lower than 80%, the present study indicate that SP+AS combination slowed the escalation of resistant *dhf*r alleles relative to Kilombero-Ulanga, from 11% higher in Rufiji in 2002 to 1% lower in 2006 relative to Kilombero-Ulanga, even though the SP cure rate was lower than 51% in the population [33].

While the growth of triple mutant allele in Kilombero-Ulanga can be explained by the expansion of the triple mutant *dhfr *lineage under selection by SP treatment, the failure of SP+AS combination to halt the increase of triple mutant *dhfr *allele frequency in Rufiji requires some explanations. There are several key ways in which the application of drug pressure solely through provision of SP+AS through government health facilities could have been undermined. Firstly, in Tanzania the availability of drugs is unregulated and varieties of anti-malarial drugs were widely available in shops and kiosks for self-medication during the time of this study [36]. Secondly, the unmatched half-life of 5-10 days for the SP and 45 minutes for the AS leaves little chance for two component drugs to work synergistically as a combination. Consequently, any new infection acquired after AS is cleared in the blood will likely be exposed to SP monotherapy providing opportunity for SP selection. Thirdly, access to treatment was imperfect, only a proportion of infections were treated as a result of high proportions of untreated asymptomatic infections in this area of high and stable malaria transmission. In the present study, the results suggests on average 20% of healthy looking individuals were infected with *P. falciparum *malaria without expressing clinical symptoms of the disease in Rufiji (see Table 2). This provides a very significant reservoir of parasite populations that are not exposed to chemotherapy [37,38]. The effect of the reservoir is that, it provides refugia for parasites which are untreated which compete with those exposed to ACT. Fourthly, by the time ACT begun in Rufiji in late 2002, the *dhfr *triple mutant allele frequency had risen to approximately 63% compromising the possibility of mutual protection of the component drugs in the ACT, the SP and AS. Lastly, While National treatment policy change was found to have a significant major effect on the frequency of resistance alleles, the switch to ACT was applied in one district only, and genetic exchange with surrounding areas which were still subject to selection by SP monotherapy may have contributed to the dilution of the effects of the intervention. This suggestion is supported by studies using *dhfr *flanking micosatellite [15] indicating that once established resistant *dhfr *alleles are highly mobile and this observation was confirmed using similar microsatellite markers in the present study.

Alleles with intermediate resistance, double *dhfr *mutant N51I+S108N and C59R+S108N did not seem to respond to the strong selection pressure exerted by intensified SP use in Kilombero-Ulanga nor to the apparently reduced SP pressure by the use of ACT in Rufiji, presenting with a constant frequency of 10% throughout in both populations. Identification of mutation at codon 164 of *dhfr *is a matter of great concern. This novel resistant allele occurred as a minority mixture in majority sensitive *dhfr *allele and in only a single isolate in this study, suggesting that this highly resistant *dhfr *genotype allele is beginning to appear but presently, it is still very rare. In the previous study conducted on the same area, around 7,000 *P. falciparum*-positive isolates from both symptomatic and asymptomatic patients were genotyped but none contained the mutant I164L *dhfr *mutation. These findings corroborate recent similar findings in different malaria endemic areas of east Africa, Muheza-Tanzania [10], Uganda [11,14], Malawi [12], and Kenya [13], which reported the I164L mutation at a low frequency, and warn of increasing appearance of this highly resistant *P. falciparum *in Africa. Most encouraging perhaps is the fact that, currently most of East African countries have already replaced SP with artemisinin-based combination therapies (ACT) as first-line anti-malarial treatment in their respective national guidelines. This will markedly reduce SP usage, which is the key determinant of its selection strength hence limiting either further selection of I164L mutation or establishment of its dispersal mechanism in the population.

Assessment of the evolutionary origin of the resistant *dhfr *alleles associated with pyrimethamine resistance in *P. falciparum *isolates from an area of high malaria transmission in two districts of south eastern part of Tanzania has confirmed some previous findings, but also generated new observations which are important for understanding the evolution of resistance at *dhfr *in African populations. The study detected high genetic diversity in the sensitive *dhfr *allele supporting the view that this is the ancestral form of *dhfr *[15,16]. This finding is consistent with previous studies elsewhere [13,14,16-20,30]. The predominant triple mutant *dhfr *flanking microsatellite haplotype observed in this study (H90: 108 bp/176 bp/203 bp) was identical to the Asian type haplotype reported in [16] and subsequent studies indicated it is widespread in Africa [13,14,17-20,39,40].

The findings of this study shows that, 25% of triple mutant *dhfr *flanking microsatellite haplotypes shared similar allele length with haplotype H90: 108 bp/176 bp/203 bp at one or two microsatellite loci, indicating that the variation observed are possibly explained by the evolution of H90: 108 bp/176 bp/203 bp through replication errors (mutation) [32] or recombination as reported in [15] rather than because of a local independent emergence. Two haplotypes (H29: 87 bp/174 bp/207 bp and H58: 87 bp/184 bp/199 bp), had completely different allele length at all three microsatellite loci and exhibited variation by 1 or 10 repeats compared to haplotype H90: 108 bp/176 bp/203 bp, and in this case it suggests they have arisen independently. Both haplotypes were carrying an allele which is characteristically associated with the *dhfr *double mutant at the closest microsatellite (-0.3 Kb) loci, and this indicates that these haplotypes may have arisen through recombination between *dhfr *double mutant and *dhfr *triple mutant flanking sequences after the unique *dhfr *haplotype H90: 108 bp/176 bp/203 bp had undergone series of replication errors at the -4.4 Kb and -5.3 Kb loci leading to the 1 or 2 repeat variations observed at the two distant microsatellite loci.

Confirmation of this interpretation may require genotyping of these isolates at other polymorphic sequences that are closely linked to the *dhfr *gene. Assessment of the *dhfr *double mutant flanking microsatellite sequences revealed intermediate levels of diversity with sequence variation being more conserved than the sensitives but more diverse than the triple mutant. The two *dhfr *double mutant alleles (C59R+S108N and N51I+S108N), each had four different common haplotypes indicating that each has arisen independently at least four times, consistent with previous reports [15-17]. A caution however, has to be taken when interpreting haplotype H51: (87 bp/182 bp/193 bp) which was common to both C59R+S108N and N51I+S108N *dhfr *double mutant alleles. This haplotype was the most common for the N51I+S108N *dhfr *double mutant allele in the present study and is consistent with earlier findings in the Tanzanian parasite population [15]. It could be a result of mutation, recombination or both, involving the two distant loci (-4.4 Kb and -5.3 Kb) of the C59R+S108N *dhfr *double mutant flanking microsatellite sequences. Generally, there was a high degree of sharing of both allele length at different microsatellite loci among the *dhfr *double and triple mutant alleles, highlighting high levels of recombination and errors of replication associated with extreme malaria transmission pattern of the study area. The high level of recombination detected in our study is consistent with recent finding in Kilifi Kenya [20], likely reflecting high malaria transmission in the eastern African zone.

Taken together, data from this study show that all 184 *dhfr *triple mutant isolates typed (perhaps with 2 possible exceptions) are related to the Asian ancestral type previously described in [15] and later found to be dispersed widely in Africa [13,14,39,40]. A study in Western Kenya has also found additional *dhfr *triple mutant novel haplotypes suggesting multiple origin of the *dhfr *triple mutant allele [13]. Further studies may be necessary to explore the possibility of replication of Kenyan findings. The findings of the present study however, is very similar to reports of recent studies in Uganda [14], Kenya [20] and that of Maiga and co-workers involving samples from 11 sub-Saharan African countries with different levels of malaria transmission [19]. All these studies identified a common evolutionary history of the *dhfr *triple mutant allele most likely indicating that this unique lineage is responsible for antifolate resistance throughout African region.

Theory suggests SP drug pressure is the driving force behind evolution of its resistance. Positive selective pressure act to increase the frequency of favoured allele meanwhile creating association with the sequences immediately flanking the gene. Initially there is generalized association but as frequency of the favoured allele increases recombination breaks down the more distant associations retaining only associations of the allele with sequences immediately flanking it, the hitchhiker [41,42]. Ultimately, the signature of selection is a pattern of reduced gene diversity or expected heterozygosity, He and the loss of diversity is described as selective sweep.

In the present study, the progression of triple mutant *dhfr *allele frequency decreased from 11% higher in Rufiji in 2002 to 1% lower in 2006 relative to Kilombero-Ulanga, clearly indicating reduction of SP drug pressure by the ACT application in Rufiji. Comparison of heterozygosity (He) values for the triple mutant *dhfr *allele (C59R+N51I+S108N) after four years of SP+AS implementation in Rufiji and four years of SP monotherapy in Kilombero-Ulanga revealed a clear signature of SP selection in Kilombero-Ulanga and a recovery of loss of gene diversity in Rufiji. The diversity values dropped from 0.65 to 0.23 in Kilombero-Ulanga population between 2001/02 and 2006 while in Rufiji the diversity around the allele recovered from 0.34 to 0.55 between 2001/02 and 2006 indicating that, the ACT attributable reduction of SP selection pressure allowed the recovery of gene diversity values through recombination (Figures 5 and 6). The observed significant reduction of gene diversity in Kilombero-Ulanga where SP selection was taking place since 2001 is consistent with previous reports in parasites from south east Africa showing significant loss of diversity across a region of 70 kb around the triple mutant *dhfr *allele as an evidence of a selective sweep attributable to selection through widespread use of SP for the treatment of malaria [17].

Finally, like most of preceding studies, the present study has also shown that pyrimethamine resistance is driven by the expansion of the unique Asian triple mutant *dhfr *allele lineage and recombination and errors of replication were largely responsible for the erosion of this lineage. This, therefore, emphasizes the role of gene flow in the dispersal of parasite population as suggested earlier [15,17] calling for continental policies to control the drug resistant malaria.

## Competing interests

The authors declare that they have no competing interests.

## Authors' contributions

ALM: Participated in study design, carried out the molecular genotyping, statistical analysis, interpretation of the data and drafting of the manuscript. RP: Participated in molecular genotyping, statistical analysis, interpretation of the data and critical review of the manuscript. BM: Participated in the data interpretation and critical review of the manuscript. SA and PK: Oversaw all aspects of the study, including design and execution of the field work, analysis and interpretation of the data and critical review of the manuscript. HM: Participated in the conception and designing of the study and critical review of the manuscript. PB: Original conception and designing of the study and critical review of the manuscript. CR: Conception of the study, oversaw all the molecular aspect of the study, participated statistical analysis, interpretation of the data and drafting of the manuscript. All the authors read and approve final manuscript for submission

## Supplementary Material

Additional file 1 **Flanking *dhfr *microsatellite polymorphisms detected in two cross sectional surveys of 2001/2002 and 2006 in Rufiji and Kilombero-Ulanga A) Sensitive allele (N51, C59, S108) B), Double mutant (C59R, S108N) allele, C) Double mutant (N51I, S108N) allele and D) Triple mutant (N51I, C59R, S108N) allele**. Click here for file
