# Supplementary material for: The evolution of pyrimethamine resistant dhfr in Plasmodium falciparum of south-eastern Tanzania: comparing selection under SP alone vs SP+artesunate combination
Source: Malar J. 2011 Oct 26;10:317. doi: 10.1186/1475-2875-10-317 (PMC3213132; doi:10.1186/1475-2875-10-317)
Supplement: Additional file 1 — Flanking dhfr microsatellite polymorphisms detected in two cross sectional surveys of 2001/2002 and 2006 in Rufiji and Kilombero-Ulanga A) Sensitive allele (N51, C59, S108) B), Double mutant (C59R, S108N) allele, C) Double mutant (N51I, S108N) allele and D) Triple mutant (N51I, C59R, S108N) allele. [file 1475-2875-10-317-S1.DOC]

**Additional file 1**

Flanking *dhfr* microsatellite polymorphisms detected in two cross sectional surrveys of 2001/2002 and 2006 in Rufiji and Kilombero-Ulanga A) Sensitive allele (N51, C59, S108) B), Double mutant (C59R, S108N) allele, C) Double mutant (N51I, S108N) allele and D) Triple mutant (N51I, C59R, S108N) allele.

1. **Sensitive allele (N51, C59, S108)**

| **Haplotype** | **n** | **-0.3** | **-4.4** | **-5.3** |
| --- | --- | --- | --- | --- |
| H4 | 1 | 87 | 158 | 205 |
| H5 | 1 | 87 | 158 | 207 |
| H6 | 1 | 87 | 158 | 215 |
| H7 | 1 | 87 | 160 | 199 |
| H10 | 1 | 87 | 164 | 211 |
| H12 | 1 | 87 | 166 | 209 |
| H16 | 2 | 87 | 170 | 193 |
| H17 | 1 | 87 | 170 | 197 |
| H19 | 2 | 87 | 170 | 207 |
| H20 | 1 | 87 | 170 | 211 |
| H21 | 2 | 87 | 170 | 213 |
| H22 | 3 | 87 | 172 | 205 |
| H23 | 2 | 87 | 172 | 207 |
| H24 | 1 | 87 | 172 | 219 |
| H25 | 1 | 87 | 174 | 190 |
| H26 | 1 | 87 | 174 | 195 |
| H28 | 2 | 87 | 174 | 201 |
| H30 | 1 | 87 | 174 | 211 |
| H31 | 1 | 87 | 176 | 191 |
| H32 | 1 | 87 | 176 | 193 |
| H35 | 8 | 87 | 176 | 203 |
| H36 | 3 | 87 | 176 | 205 |
| H37 | 2 | 87 | 176 | 207 |
| H39 | 2 | 87 | 178 | 193 |
| H40 | 1 | 87 | 178 | 197 |
| H41 | 1 | 87 | 178 | 199 |
| H42 | 1 | 87 | 178 | 207 |
| H47 | 1 | 87 | 180 | 203 |
| H48 | 2 | 87 | 180 | 205 |
| H49 | 2 | 87 | 180 | 207 |
| H50 | 1 | 87 | 180 | 213 |
| H51 | 1 | 87 | 182 | 193 |
| H53 | 1 | 87 | 182 | 205 |
| H55 | 1 | 87 | 182 | 209 |

**Sensitive allele (N51, C59, S108) continued**

| **Haplotype** | **n** | **-0.3** | **-4.4** | **-5.3** |
| --- | --- | --- | --- | --- |
| H59 | 1 | 87 | 184 | 201 |
| H60 | 2 | 87 | 184 | 207 |
| H62 | 1 | 87 | 184 | 215 |
| H65 | 1 | 87 | 186 | 205 |
| H67 | 1 | 87 | 188 | 209 |
| H68 | 1 | 87 | 193 | 193 |
| H69 | 1 | 87 | 193 | 195 |
| H71 | 1 | 92 | 166 | 199 |
| H72 | 1 | 96 | 178 | 205 |
| H73 | 1 | 96 | 180 | 207 |
| H74 | 1 | 96 | 180 | 211 |
| H75 | 1 | 96 | 182 | 199 |
| H76 | 1 | 98 | 178 | 215 |
| H77 | 1 | 100 | 174 | 213 |
| H79 | 1 | 102 | 172 | 217 |
| H80 | 1 | 102 | 176 | 201 |
| H82 | 1 | 104 | 168 | 203 |
| H86 | 1 | 108 | 172 | 195 |
| H98 | 1 | 110 | 158 | 191 |
| H99 | 2 | 110 | 158 | 205 |
| H101 | 1 | 110 | 184 | 197 |
| H104 | 1 | 115 | 184 | 199 |

1. **Double mutant *dhfr* (C59R, S108N) allele**

| **Haplotype** | **n** | **-0.3** | **-4.4** | **-5.3** |
| --- | --- | --- | --- | --- |
| H3 | 1 | 87 | 156 | 203 |
| H8 | 1 | 87 | 162 | 199 |
| H9 | 1 | 87 | 164 | 199 |
| H14 | 2 | 87 | 168 | 199 |
| H15 | 1 | 87 | 168 | 205 |
| H22 | 1 | 87 | 172 | 205 |
| H27 | 1 | 87 | 174 | 199 |
| H32 | 2 | 87 | 176 | 193 |
| H34 | 18 | 87 | 176 | 199 |
| H35 | 10 | 87 | 176 | 203 |
| H39 | 1 | 87 | 178 | 193 |
| H45 | 1 | 87 | 180 | 193 |
| H51^a^ | 6 | 87 | 182 | 193 |
| H52 | 1 | 87 | 182 | 199 |
| H54 | 1 | 87 | 182 | 207 |
| H64 ^a^ | 27 | 87 | 186 | 199 |
| H65 | 1 | 87 | 186 | 205 |
| H66 | 1 | 87 | 188 | 199 |
| H102 | 1 | 110 | 186 | 199 |

Reported previously ^a^[15]

**C) Double mutant *dhfr* (N51I, S108N) allele**

| **Haplotype** | **n** | **-0.3** | **-4.4** | **-5.3** |
| --- | --- | --- | --- | --- |
| H2 | 1 | 85 | 184 | 190 |
| H11 | 1 | 87 | 166 | 193 |
| H13 | 3 | 87 | 168 | 193 |
| H16 | 1 | 87 | 170 | 193 |
| H22 | 5 | 87 | 172 | 205 |
| H32 | 6 | 87 | 176 | 193 |
| H33 | 1 | 87 | 176 | 198 |
| H35 | 2 | 87 | 176 | 203 |
| H36 | 1 | 87 | 176 | 205 |
| H39^b^ | 19 | 87 | 178 | 193 |
| H43 | 1 | 87 | 178 | 209 |
| H44 | 1 | 87 | 178 | 211 |
| H45 | 3 | 87 | 180 | 193 |
| H46 | 1 | 87 | 180 | 201 |
| H47 | 2 | 87 | 180 | 203 |
| H51 ^a^ | 44 | 87 | 182 | 193 |
| H52 | 2 | 87 | 182 | 199 |
| H53 | 1 | 87 | 182 | 205 |
| H56 | 1 | 87 | 184 | 193 |
| H57 | 1 | 87 | 184 | 199 |
| H61 | 1 | 87 | 184 | 209 |
| H63 | 2 | 87 | 186 | 193 |
| H70 | 1 | 87 | 199 | 207 |
| H89 | 2 | 108 | 176 | 193 |
| H90 | 2 | 108 | 176 | 203 |
| H91 | 1 | 108 | 176 | 205 |

Reported previously ^a^[15], ^b^[14]

**D) Triple mutant *dhfr* (N51I, C59R, and S108N) allele**

| **Haplotype** | **n** | **-0.3** | **-4.4** | **-5.3** |
| --- | --- | --- | --- | --- |
| H1 | 1 | 85 | 176 | 203 |
| H18 | 1 | 87 | 170 | 203 |
| H29 | 1 | 87 | 174 | 207 |
| H32 | 3 | 87 | 176 | 193 |
| H35 | 21 | 87 | 176 | 203 |
| H38 | 1 | 87 | 176 | 211 |
| H58 | 1 | 87 | 184 | 199 |
| H78 | 1 | 102 | 162 | 203 |
| H81 | 1 | 102 | 176 | 203 |
| H83 | 1 | 104 | 176 | 203 |
| H84 | 1 | 108 | 166 | 203 |
| H85 | 1 | 108 | 170 | 205 |
| H87 | 1 | 108 | 172 | 199 |
| H88 | 1 | 108 | 172 | 203 |
| H89 | 2 | 108 | 176 | 193 |
| H90^a^ | 135 | 108 | 176 | 203 |
| H92 | 1 | 108 | 176 | 207 |
| H93 | 1 | 108 | 178 | 201 |
| H94 | 2 | 108 | 178 | 203 |
| H95 | 1 | 108 | 178 | 207 |
| H96 | 1 | 108 | 182 | 221 |
| H97 | 1 | 108 | 186 | 203 |
| H100 | 3 | 110 | 176 | 203 |
| H103 | 1 | 115 | 176 | 203 |

Reported previously ^a^[15]
